# Quality of Calcium Food Supplements: Evaluation Compared to Manufacturers’ Declarations

**DOI:** 10.3390/molecules27238154

**Published:** 2022-11-23

**Authors:** Anna Puścion-Jakubik, Gabriela Staniaszek, Patrycja Brzozowska, Katarzyna Socha

**Affiliations:** Department of Bromatology, Faculty of Pharmacy with the Division of Laboratory Medicine, Medical University of Białystok, Mickiewicza 2D Street, 15-222 Białystok, Poland

**Keywords:** food supplements, calcium, osteoporosis, skin, hair and nails, nutricosmetics, allergy

## Abstract

Calcium (Ca) is a macronutrient necessary for the proper functioning of an organism. In the case of insufficient consumption with diet, its deficiencies can be supplemented with food supplements (FS). These supplements are used, for example, as an auxiliary in the prevention of osteoporosis, allergies, hair loss or nail brittleness. The purpose of the study was to assess the compliance of Ca content with the manufacturers’ declaration. The material consisted of 108 FS. Ca content was determined by atomic absorption spectrometry (AAS). It was shown that 1.9% of the samples were characterized by a Ca content that was too low in comparison to the manufacturer’s declaration, while a content that was relatively too high was found in 54.6% of FS. The quality of FS should be monitored to ensure patient safety.

## 1. Introduction

Food supplements (FS) are foods that are intended to supplement a normal diet. FS are a concentrated source of nutrients or other substances that have a nutritional or physiological effect. Supplements can contain one ingredient or more. They are available for sale in a form that allows patients to precisely dose (e.g., tablets, capsules, sachets) [1].

The use of dietary supplements is very widespread. According to the report “Poles and dietary supplements”, over 67% of Poles declare the use of supplementation, and nearly 76% emphasize that they use it regularly. The most frequently taken preparations included vitamin and mineral supplements (81.1%), supplements to improve the appearance of skin, hair and nails (33.2%) and supplements to strengthen joints and bones (31.4%). These FS categories may include, inter alia, calcium (Ca). Worryingly, over 45% of consumers do not consult any specialist on purchasing supplements [2,3].

In recent years, research in different countries on FS has focused mainly on quality assessment, including the determination of the content of impurities such as mercury [4,5], arsenic [6], cyanotoxins [7], polycyclic aromatic hydrocarbons [8], as well as drug components that should not be present in FS, such as potency substances [9] and pharmaceutical adulterants, such as anxiolytics, antidepressants, anorexics, stimulants and laxatives [10]. A significant problem that is not addressed in scientific research is incorrect content of the declared ingredients in FS.

Despite the widespread use of FS, it should be emphasized that their quality is not sufficiently controlled. A comprehensive assessment of the quality of the FS market in Poland was carried out by the Supreme Chamber of Control in 2017–2020. Its aim was to assess the safety of placing FS on the market and trading them. The summary report shows that due to the lack of an appropriate monitoring system, consumers may have been exposed to inadequate quality preparations for many years. This is primarily related to the intensive development of the supplement industry and the insufficient capacities of the regulatory authorities. From 2017 to 2020, only in Poland itself, over 62,000 notifications of the first marketing of FS were submitted. With regard to approximately 56,000 preparations, the verification process was not undertaken at all. In some cases, the verification process took several years, and the analyzed preparations could still be available for sale [11]. An important aspect is also the discrepancy between the declared and marked content. This research problem is not addressed sufficiently in publications.

Ca is a mineral that is essential for the functioning of the human organism. In an adult, it constitutes about 2% of body weight, which is on average about 1200 g [12]. Ca plays a major role in the conduction of nerve stimuli, hormonal regulation, muscle contractility and the activation of certain enzymes [13].

According to the legislation, 15 minerals, including Ca, can be contained in FS. Ca may be present in the following combinations: Ca carbonate, Ca chloride, Ca gluconate, Ca glycerophosphate, Ca hydroxide, Ca lactate, Ca oxide, Ca salts of citric acid and Ca salts of orthophosphoric acid [1]. Pursuant to the resolution of the Committee for Dietary Supplements, operating at the Sanitary and Epidemiological Council, the maximum content of Ca in the recommended daily dose of FS intended for consumption by adults is 1500 mg [14]. This is based on the European guidelines, which indicate the Maximum Supplement Level (MSL) [15]. The permissible deviation of the content of the mineral component is from −20% to 45% declared value [16,17].

Therefore, the objective of this study was to assess the Ca content in various FS available on the market in Poland in relation to the manufacturers’ declarations regarding the Ca content.

## 2. Results

The results regarding the Ca content in selected groups of FS, taking into account several classification criteria, are presented in Table 1, Table 2, Table 3, Table 4, Table 5, Table 6, Table 7 and Table 8. The results were presented as one serving or one dose (1 tablet, 1 capsule etc.). The differences in the doses taken during the day were converted into daily doses (taking into account the number of doses recommended for consumption during the day).

Considering the pharmaceutical form, the tablets were characterized by the highest median Ca content (Table 1), this amount was significantly higher compared to the Ca content in the other pharmaceutical forms of FS (383.1 vs. 74.6 mg/dose).

Another criterion used was the price. Three subgroups were adopted: cheap preparations (price below PLN 10), medium-price preparations (PLN 10–20) and high-price preparations (over PLN 20), taking into account the average earnings in Poland. The highest median Ca content was shown in preparations with an average price, between PLN 10 and PLN 20. However, the median Ca content was not higher compared to the cheaper and more expensive preparations (Table 2).

Another criterion for the division was the age of the patients for whom the FS were intended (Table 3). The smallest percentage of preparations on the market were supplements intended only for children; their Ca content was significantly lower compared to the Ca content in FS intended for both children and adults (89.6 vs. 259.8 mg/dose), as well as when compared to preparations which were dedicated only to adults (89.6 vs. 254.9 mg/dose).

The next division criterion was the country of origin of the producer producing a supplement (Table 4). The vast majority of supplements came from the domestic market (*n* = 81). The highest median Ca content was found in the preparations from Japan (596 mg/dose), but these values were not statistically significant.

Taking into account the chemical form, the highest percentage of FS contained Ca carbonate (72 out of 108 preparations). Supplements with this form had the highest median Ca content (335.4 mg/dose), but this difference was not statistically significant compared to other chemical forms (Table 5).

Supplements with a Ca content declared by the producers to be at the level of up to 100 mg/dose had a significantly lower median content compared to preparations with the content of 100 to 200 mg (95.3 vs. 208.2 mg/dose). The median Ca content for the preparations declared above 200 mg was 521.0 mg/dose (Table 6).

**Figure 1:**
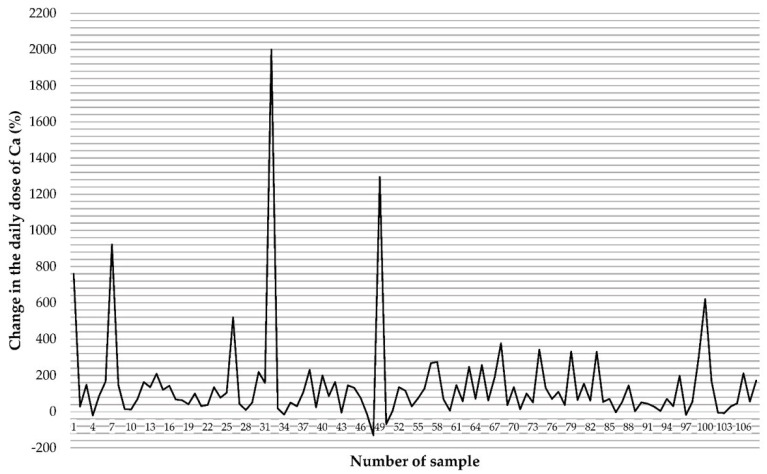
The disparity between the declared Ca content and that determined in food supplements based on the daily dose consumed.

Preparations containing only one mineral component (Ca) were characterized by a higher median compared to multi-component preparations, but these differences were not statistically significant (Table 7). Taking the determined Ca content in individual FS and the number of servings recommended for consumption during the day, we calculated that patients can consume up to 130% less than the expected content (in this preparation, the declared value is 406 mg/dose, the marked value is 141 mg/dose, thus by consuming two doses a day, the patient consumes 530 mg less than they should) (Figure 1). Due to the very large dispersion and the need to improve the legibility of the figure, the preparation with the highest positive dispersion was symbolically marked as a 2000% dispersion but the actual dispersion was many times greater: the declared value is 0.12 mg/dose, while the marked value is 103 mg, which means that the patient takes as much as 85,653% more Ca than it appears from the declaration on the packaging.

The analysis of the Ca content in FS, in comparison to the applicable norms of the spread of the values of the results, showed that 1.9% of the samples had a too low Ca content in relation to the declaration, while in 54.6% of the supplements the content of the tested element was found to be too high.

We also assessed which subcategory of supplements was characterized by the highest percentage of samples with the standard (Table 8), and these were effervescent tablets (among all tested supplements, effervescent tablets within the normal range constituted 21.30% of the samples), with a price below PLN 10 (20.37%), for adults (31.48%), from Poland (35.18%), containing Ca carbonate (26.85%), with Ca content between 100 and 200 mg (25.00%), and complex preparations (36.11%).

## 3. Discussion

The research conducted as part of this project assessed the quality of 108 FS available for sale in Poland. These were preparations characterized by a different composition, price, pharmaceutical form or origin. The analysis of Ca content in one portion showed that most of the preparations contain more Ca than declared by the declarations placed by the producers on the packaging (54.6%). A small percentage of the samples was characterized by a Ca content lower than allowed by the standards (1.9%). We showed that 43.5% of the preparations were within the acceptable standards.

The problem of the difference between the value placed on the package and that determined by analytical methods in FS containing Ca has not been discussed in scientific publications so far; therefore, the following considerations are speculative.

A Ca content that is higher by several dozen or several hundred percent in one portion of the FS compared to the declared value, if used chronically, may lead to side effects. A retrospective study by Machado et al. in 2015 aimed at assessing the frequency of hypercalcemia associated with the use of supplementation with Ca supplements. Hypercalcemia was found in 429 patients over 18 years of age who were admitted to hospital between 2010 and 2013. The study excluded those patients whose hypercalcemia was caused by primary hyperparathyroidism, cancer, sarcoidosis or other diseases. Of 72 patients with hypercalcemia unrelated to PTH, 15 (20.8%) met the criteria for the diagnosis of Ca supplementation syndrome (renal failure, metabolic alkalosis, elevated serum bicarbonate and taking Ca/vitamin D supplements). Out of these 15 patients, 12 people reported complaints and symptoms, the cause of which was hypercalcemia (weakness, polyuria, abdominal pain, constipation, mental disorders and musculoskeletal discomfort). All patients received intravenous hydration, discontinued FS, and six patients received pharmacological treatment that lowered Ca levels in the form of calcitonin or zoledronic acid. Supplementation of Ca and vitamin D is considered beneficial for general health and prophylaxis, and also advised in the treatment of osteoporosis and vitamin D deficiency. However, it should be emphasized that the indiscriminate use of Ca and vitamin D supplements may have numerous health consequences [18]. The literature describes the case of a 36-year-old woman who developed severe hypercalcemia a few days after starting to breastfeed her second child. During and after pregnancy, the woman supplemented her high-calcium diet with moderate amounts of Ca carbonate to avoid an osteoporotic fracture that she had experienced while breastfeeding her first child. Metabolic changes occurring during lactation predispose women to hypercalcemia, therefore the recommended daily intake of Ca during breastfeeding should not be exceeded [19].

The highest Ca content determined in one portion of the FS is 2610 mg, while the maximum Ca content in the recommended daily portion in FS dedicated to adults is 1500 mg in accordance with the Resolution of the Team for Diet Supplements [14]. All other FS delivered less than 1500 mg per serving.

**Figure 2:**
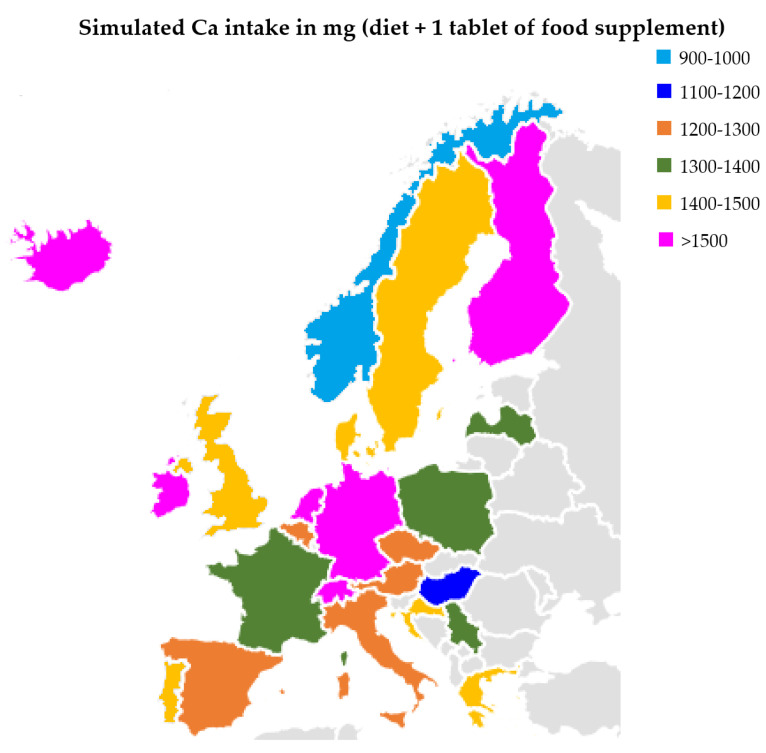
Calcium intake in selected European countries (with diet and 1 tablet of Ca).

The European Food Safety Authority (EFSA) determined the population reference intake for Ca women and men aged 25 to 50 to be at the level of 950 mg [20]. Research published in 2017 indicated that the average Ca intake is 800–900 mg in Poland, 900–1000 mg in Croatia, and over 1000 mg in Germany [21], for example. Our research has shown that the most popular pharmaceutical form of Ca present on the market are tablets. The average content in 1 serving (1 tablet) is 498.8 ± 407.0 mg. Assuming that Polish residents will use 1 tablet each, their Ca intake will increase to 1300–1400 mg, and in the case of German residents it will be at least 1500 (Figure 2). It should be emphasized that for some supplements it is recommended to take at least 2 tablets a day. A separate issue is Ca intake in children. For example, the EFSA recommendation for children aged 4–5 is 800 mg. The average content of children’s supplements was 105.5 ± 88.0 mg. In the case of this group, all preparations were characterized by a lower Ca content than the declared value.

Patients expect health benefits when buying FS and compliance of the declared value on the packaging with the actual content in the accepted FS. In the case of a deficiency diet supplemented with a preparation with a low content of Ca, nails may become brittle, the skin may become dry, and confusion can occur among the elderly. In turn, hypercalcemia can result in constipation, kidney stones and mood changes. Although the considerations regarding the Ca content in FS to predict health outcomes in the population are speculative, they constitute an important area of research.

The requirement for Ca varies depending on the age group. The highest amounts should be consumed during the period of rapid growth and puberty—the norm for girls and boys aged 10 to 18 at the estimated average requirement (EAR) level is 1100 mg/day—and in the elderly (1000 mg/day for people over 66 years of age). Studies have not found any additional benefits of consuming Ca in amounts greater than the recommended values [22]. Of the FS tested, 7.4% contained more than 1000 mg Ca/portion.

The average dietary intake of Ca in adults is at the level of 60% of the normal coverage [22]. It is related to the low absorption of Ca from diet (about 25%). Ca is best absorbed from milk and its products, while the lowest absorption of Ca is characteristic of plant products, mainly due to the presence of oxalic acid and phytic acid. Additionally, the intake of this element from diet is hindered by the presence of insoluble fractions of dietary fiber, fat and too high phosphorus content [23]. If it is not possible to cover the demand for this element with the diet, it is recommended to use supplementation.

Ca deficiency leads to, among other things, an increased risk of osteoporosis. Other health consequences include increased excitability of the organism, tetany, neurological disorders and an increase in blood pressure [24]. Therefore, more and more people are supplementing Ca deficiencies with FS. However, it should be emphasized that, compared to drugs, FS are not subject to sufficient control. There are no legal regulations that require testing of the quantitative and qualitative composition of FS before being approved for sale. These preparations are subject to standard food control tests, but only after they have been released for sale and to a limited extent. This means that consumption of a food supplement, despite the declared content of minerals, does not ensure that the potential deficiencies in the diet will be supplemented [11]. Moreover, the tolerance limits for FS, taking into account the uncertainty in the measurement, are quite wide: for vitamins it is from −20% to +50%, and for minerals from −20% to +45% [1].

The current norms regarding the recommended daily intake of Ca for the population of Poland over 19 years of age require the consumption of 1000 mg of this ingredient [22]. According to a study conducted by EFSA in 2015, the average Ca intake among people over 18 in nine European countries ranged from 690 mg to 1122 mg per day [25]. An American study covering the years 2009–2010 showed that the daily Ca intake among people aged 20–39 was 1210 mg for men and 947 mg for women [26]. In contrast, a study conducted in Poland in the years 2003–2005 on 1855 people aged from 20 to 34 showed that the average daily consumption of Ca in this group was 696 mg in men and 518 mg in women [27]. According to the results of a study from 2013–2014, which was also carried out in Poland on a group of people over 20 years of age, the average consumption of Ca for men was 586 mg/day and for women was 523 mg/day [28]. The above-mentioned examples show that the consumption of Ca in the Polish population is insufficient. In the case of insufficient amounts of this element in the diet, supplementation with appropriately high-quality preparations seems necessary.

Slightly different conclusions were formulated based on the assessment of Ca intake, carried out in a group of women (*n* = 593) aged 18–50 practicing sports at least twice a week. The standard for Ca was 800 mg. The median intake of Ca in the study group was 502 mg/day. As many as 92.0% of the respondents had a consumption of this ingredient below the norm at the EAR level. The authors estimated that 13.1% of women used Ca supplementation, but as many as 11.5% did not need an additional supply of this important mineral [29].

Over the years, many papers have been published, including systematic reviews and meta-analyses, evaluating the role of Ca for example in prevention of colorectal cancer [30], osteoporosis [31] and cardiometabolic disorders [32].

Currently, the expert consensus on Ca concerns, inter alia, reveals the following issues: supplementation with Ca and vitamin D results in a slight reduction in the risk of fractures, while supplementation with Ca alone is not effective. However, intervention at the level of whole populations has not been shown to be an effective strategy from a public health perspective. It is emphasized that side effects resulting from Ca supplementation may include the occurrence of gastrointestinal symptoms and kidney stones, while the issue of an increased risk of cardiovascular events requires further research. It should be added that supplementation with vitamin D alone is more effective in reducing the risk of falls. Contemporary recommendations indicate the need for calcium and vitamin D supplementation in patients at high risk of deficiency of these components and in patients treated for osteoporosis [33].

In the case of health consequences resulting from improper supplementation, pharmacokinetic factors should also be considered. Ca can be absorbed from the intestinal lumen into the blood by transcellular and paracellular routes. The transcellular pathway is an active process, taking place, for example, in the duodenum and jejunum. The paracellular pathway is a passive process that occurs in the ileum and jejunum [34] among others.

Changes in the bioavailability of Ca during food storage were assessed, among others, in Ca compounds added to lemon juice. The highest bioavailability at the start of the study and after 6 months was found in calcium amino acid chelate (45.09 ± 0.59% and 45.57 ± 2.12%, respectively), Ca pidolate (38.09 ± 0.28% and 34.38 ± 1.13%, respectively), Ca lactate (32.4 ± 2.17% and 32.68 ± 1.27%, respectively) and Ca triphosphate (31.21 ± 4.43% and 30.48 ± 0.32%, respectively) [35].

A puzzling aspect is why manufacturers add higher Ca content to FS than declared. It seems necessary to introduce routine analysis of preparations, because currently this issue is not sufficiently controlled. As emphasized above, long-term supplementation with preparations with a higher content of Ca than the declared value can sometimes be harmful.

Among the limitations of this study, the following aspects could be mentioned: the quality assessment was carried out on the most popular preparations available for sale in pharmacies; therefore, the quality of preparations available, for example, in shops, herbal medicine shops, etc., may be different. In addition, the advantage of certain categories may also be indicated as a limitation, and not a homogeneous distribution, e.g., by far the largest percentage was for effervescent tablets and tablets, preparations typically aimed at adults or containing Ca carbonate in the composition. Further research should focus on the assessment of the bioavailability of the various chemical forms of Ca, and on the assessment of the concentration obtained after consumption of FS. Moreover, it seems necessary to evaluate the changes in Ca content during FS storage. In addition, due to the need to develop research on calcium absorption from FS, this discussion is speculative and indicates the direction of further analyses.

## 4. Materials and Methods

### 4.1. Materials

FS were selected on the basis of a review of the assortment available in stationary and online pharmacies, both in private pharmacies and chain pharmacies popular throughout the country. In total, 108 FS were included in the study, differing in pharmaceutical form, chemical form, producer’s country of origin, price, composition, Ca content declared by the producer and the age group to which they are intended. Three sub-samples were taken from each supplement, from three different batches.

### 4.2. Sample Preparation

FS were homogenized in a vibrating crusher (Testchem, Radlin, Poland), and about 0.3 g, with an accuracy of 0.001 g, of the obtained powder was weighed into Teflon vessels. Then, 4 mL of spectrally pure concentrated (69%) nitric acid (Tracepur, Merck, Darmstadt, Germany) was added to the vessels. The microwave mineralization process was carried out in a closed system (Berghof, Speedwave, Eningen, Germany), and the analytical program is characterized in Table 9. The mineralizates were transferred to polypropylene vessels using deionized water, and the final weights were noted.

### 4.3. Determination of Ca Content

The content of Ca in FS was assessed by atomic absorption spectrometry (AAS) with Zeeman background correction at a wavelength of 422.7 nm using the Z-2000 instrument (Hitachi, Tokyo, Japan) with acetylene–air flame atomization. Lanthanum chloride (1% LaCl_3_, Sigma-Aldrich, Merck, Darmstadt, Germany) was applied as a masking reagent. On the basis of the calibration curve made from an absorbance–Ca concentration system, the concentration of Ca in the tested samples of FS was calculated.

In order to assess the accuracy of the method, a control was performed on the certified reference material Simulated Diet D (LIVSMEDELS VERKET, National Food Administration, Uppsala, Sweden), and six determinations of Ca were carried out. The range of certified values was 473–547 mg/kg, and all results were obtained within the acceptable range. The coefficient of variation (V) and the accuracy (% of error) was 0.96% and 0.22%, respectively.

### 4.4. Statistical Analysis and Interpretation of Results

The Statistica 13.3 software (Tibco, Palo-Alto, Santa Clara, CA, USA) was used to conduct statistical analyses. The results are presented in the tables as the mean with standard deviation (Av. ± SD), minimum and maximum (Min-Max) values, the median (Med), the lower and upper quartile (Q1–Q3) and the interquartile range (IQR). The normality of the distribution of numerical data was evaluated using the Shapiro–Wilk, Kolmogorov–Smirnov and Lilliefors tests. Statistically significant differences between the groups were assessed using the Kruskal–Wallis ANOVA test and the Mann–Whitney U test, the level of significance was *p* < 0.05.

The obtained results regarding the Ca content in one dose of FS (e.g., in 1 tablet, capsule, etc.) were benchmarked to the recommendations of the European Commission from 2012 on establishing tolerance limits for minerals specified on the labels of FS. The Ca content of the preparation may be up to 20% lower and up to 45% higher than the declared value [16,17].

## 5. Conclusions

The conducted research has shown that more than half of Ca-containing food supplements are characterized by too high a content of this element; that is, they contain over 45% more than declared by the manufacturers. The universality of the use of supplementation among various age groups indicates that the quality of these preparations must be sufficiently high, and in accordance with the manufacturers’ declarations.

## Figures and Tables

**Table 1:** Calcium content in food supplements, taking into account the pharmaceutical form.

Pharmaceutical Form (Sign)	*n*	Ca Content (mg/dose)					
		Δ (%)					
		Av. ± SD	Min–Max	Med	Q1	Q3	IQR
Capsules (1)	6	243.6 ± 160.0	75.8–540.5	217.0	152.6	258.9	106.3
		70.5 ± 77.5	from −9.0 to 209.9	63.5	9.0	86.1	77.1
Effervescent tablets (2)	44	278.7 ± 150.1	58.3–595.1	227.0	162.2	365.7	203.5
		1992.9 ± 12,905.7	from −34.7 to 85,653.2	41.8	13.4	72.7	59.3
Powders (3)	6	588.1 ± 992.5	122.4–2610.0	177.3	141.5	300.3	158.9
		63.2 ± 114.6	from −65.2 to 275.0	47.8	2.0	71.6	69.6
Tablets (4)	49	498.8 ± 407.0	31.0–1661.3	383.1 * _4/5_	195.7	690.3	494.7
		90.0 ± 182.3	from −13.8 to 1295.5	63.1	28.8	99.0	70.2
Other form (5)	3	65.4 ± 44.5	17.1–104.1	74.6	17.1	104.6	87.5
		77.2 ± 8.3	from 70.7 to 86.5	74.3	70.7	86.5	15.8

Av.—average, IQR—interquartile range, Max—maximum value, Med—median, Min—minimum value, Q1—lower quartile, Q3—upper quartile, SD—standard deviation, * *p* < 0.05, Δ (%)—the difference between the declared value (taken as 100%) and the determined value.

**Table 2:** Calcium content in food supplements, taking into account the price.

Price (PLN)	*n*	Ca Content (mg/dose)					
		Δ (%)					
		Av. ± SD	Min–Max	Med	Q1	Q3	IQR
<10	44	278.4 ± 152.5	58.3–595.1	234.0	161.6	365.7	204.1
		49.3 ± 47.8	from −34.7 to 230.6	42.5	13.4	74.0	60.5
10–20	23	547.7 ± 583.2	102.7–2610.0	383.1	225.7	647.7	422.0
		3853.0 ± 17,833.7	from −4.9 to 85,653.2	65.8	28.3	128.0	99.7
>20	41	415.6 ± 392.3	17.1–1389.2	221.2	156.8	534.8	378.0
		57.2 ± 50.9	from −65.2 to 170.7	54.4	28.8	74.5	45.7

Av.—average, IQR—interquartile range, Max—maximum value, Med—median, Min—minimum value, Q1—lower quartile, Q3—upper quartile, SD—standard deviation, Δ (%)—the difference between the declared value (taken as 100%) and the determined value.

**Table 3:** Calcium content in the food supplements, taking into account the age group for which they are intended.

Age Group (Sign)	*n*	Ca Content (mg/dose)					
		Δ (%)					
		Av. ± SD	Min–Max	Med	Q1	Q3	IQR
Children (1)	4	105.5 ± 88.0	17.1–225.7	89.6 * _1/2, 1/3_	45.8	165.1	119.3
		57.2 ± 50.9	from −65.2 to 170.7	54.4	28.8	74.5	45.7
Children and adults (2)	20	338.7 ± 230.0	102.7–1145.2	259.8	215.7	379.8	164.1
		3853.0 ± 17,833.7	from −4.9 to 85,653.2	65.8	28.3	128.0	99.7
Adults (3)	84	413.0 ± 415.6	31.0–2610.0	254.9	156.3	518.2	361.9
		49.3 ± 47.8	from −34.7 to 230.6	42.5	13.4	74.0	60.5

Av.—average, IQR—interquartile range, Max—maximum value, Med—median, Min—minimum value, Q1—lower quartile, Q3—upper quartile, SD—standard deviation, * *p* < 0.05, Δ (%)—the difference between the declared value (taken as 100%) and the determined value.

**Table 4:** Calcium content in the food supplements, taking into account the country of the producer.

Country	*n*	Ca Content (mg/dose)					
		Δ (%)					
		Av. ± SD	Min–Max	Med	Q1	Q3	IQR
Croatia	3	327.6 ± 184.5	217.0–540.5	225.2	217.0	540.5	323.5
		34.7 ± 17.2	from 22.6 to 54.4	27.2	22.6	54.4	17.2
Germany	6	527.0 ± 539.7	154.6–1389.2	219.7	156.8	1021.6	864.7
		71.6 ± 54.8	from −3.3 to 131.5	74.9	30.7	120.6	54.8
Japan	4	678.0 ± 528.3	225.7–1294.1	596.0	237.7	1118.2	880.4
		106.8 ± 45.3	from 54.2 to 158.8	107.1	71.3	142.2	70.9
Poland	81	378.4 ± 391.8	31.0–2610.0	252.2	153.1	500.0	347.0
		1131.1 ± 9509.9	from −34.7 to 85,653.2	51.7	18.8	82.1	63.3
United States	8	264.7 ± 138.5	139.7–534.8	214.7	171.8	335.1	163.3
		25.7 ± 45.8	from −65.2 to 72.6	33.3	4.9	60.7	55.9
Other	6	376.9 ± 287.5	17.1–751.2	307.4	188.3	690.3	502.0
		58.0	from 25.8 to 72.6	63.8	50.2	71.6	21.4

Av.—average, IQR—interquartile range, Max—maximum value, Med—median, Min—minimum value, Q1—lower quartile, Q3—upper quartile, SD—standard deviation, Δ (%)—the difference between the declared value (taken as 100%) and the determined value.

**Table 5:** Calcium content in the food supplements, taking into account the chemical form of calcium.

Chemical Form	*n*	Ca Content (mg/dose)					
		Δ (%)					
		Av. ± SD	Min–Max	Med	Q1	Q3	IQR
Calcium carbonate	72	455.6 ± 428.6	31.0–2610.0	335.4	171.1	537.6	428.6
		1247.7 ± 10,087.5	from −65.2 to 85,653.2	57	20.2	84.5	64.3
Calcium lactate	9	271.3 ± 125.5	186.0–595.1	225.2	217.0	255.0	38.0
		54.4 ± 68.7	from 3.3 to 230.6	27.2	22.7	42.9	20.3
Calcium salts of orthophosphoric acid	7	176.6 ± 83.6	31.9–265.9	208.2	119.4	249.8	130.4
		41.2 ± 35.9	from −13.8 to 99.0	36.5	18.2	64.2	45.9
Tricalcium phosphate	3	159.0 ± 32.1	122.4–182.0	172.6	122.4	182.0	59.7
		32.5 ± 26.7	from 2.0 to 51.7	43.9	2.0	51.7	49.7
Other forms	6	220.1 ± 129.5	87.7–457.1	186.6	143.5	258.9	115.4
		65.2 ± 20.0	from 35.0 to 95.0	67.0	54.4	72.6	18.2
Several chemical forms	7	440.3 ± 425.8	152.6–1353.4	247.9	186.1	534.8	348.7
		89.9 ± 74.3	from 9.0 to 209.9	67.1	23.5	170.7	147.1
No declaration	4	132.5 ± 95.3	17.1–246.5	133.1	64.4	200.6	136.2
		350.0 ± 631.1	from −3.3 to 1295.5	53.8	16.8	683.1	631.1

Av.—average, IQR—interquartile range, Max—maximum value, Med—median, Min—minimum value, Q1—lower quartile, Q3—upper quartile, SD—standard deviation, Δ (%)—the difference between the declared value (taken as 100%) and the determined value.

**Table 6:** Calcium content in the food supplements, taking into account the content declared by the manufacturer.

Declaration of the Content (Sign)	*n*	Ca Content (mg/dose)					
		Δ (%)					
		Av. ± SD	Min–Max	Med	Q1	Q3	IQR
<100 (1)	16	96.9 ± 55.7	17.1–247.9	95.3 ** _1/2, 1/3_	66.5	115.5	49.1
		5496.1 ± 21,377.5	from −9.0 to 85,653.2	75.1	30.2	127.1	96.9
100–200 (2)	47	213.3 ± 80.4	100.7–595.1	208.2 *** _2/3_	167.5	249.8	82.3
		48.8 ± 48.3	from −16.0 to 230.6	40.8	17.3	67.5	50.2
>200 (3)	45	673.7 ± 451.7	141.5–2610.0	521.0	411.4	829.2	417.8
		64.4 ± 59.0	from −65.2 to 275.0	63.3	25.8	77.2	51.4

Av.—average, IQR—interquartile range, Max—maximum value, Med—median, Min—minimum value, Q1—lower quartile, Q3—upper quartile, SD—standard deviation, ** *p* < 0.01, *** *p* < 0.001, Δ (%)—the difference between the declared value (taken as 100%) and the determined value.

**Table 7:** Calcium content in food supplements, taking into account the type of preparation.

Type of Food Supplement	*n*	Ca Content (mg/dose)					
		Δ (%)					
		Av. ± SD	Min–Max	Med	Q1	Q3	IQR
Single	13	484.5 ± 670.3	87.7–2610.0	255.0	220.8	337.1	116.3
		70.3 ± 72.8	from 12.4 to 275.0	36.9	25.3	95.0	69.7
Complex	95	374.6 ± 330.3	17.1–1661.3	249.8	154.6	506.4	351.8
		970.7 ± 8781.7	from −65.2 to 85,653.2	54.4	21.7	77.2	55.5

Av.—average, IQR—interquartile range, Max—maximum value, Med—median, Min—minimum value, Q1—lower quartile, Q3—upper quartile, SD—standard deviation, Δ (%)—the difference between the declared value (taken as 100%) and the determined value.

**Table 8:** Relationship between criterions and percentage of food supplements with normal, below and above normal calcium levels.

Criterion	Groups	*n* (% of the Category)	Above Norm *n* (% of Subcategories)	In the Norm *n* (% of Subcategories)	Below Norm *n* (% of Subcategories)
**Pharmaceutical form**	Capsules	6 (5.56)	4 (66.67)	2 (33.33)	0 (0.00)
	Effervescent tablets	44 (40.74)	20 (45.45)	23 (52.28)	1 (2.27)
	Powders	6 (5.56)	3 (50.00)	2 (33.33)	1 (16.67)
	Tablets	49 (45.36)	29 (59.18)	20 (40.82)	0 (0.00)
	Other form	3 (2.78)	3 (100.0)	0 (0.00)	0 (0.00)
**Price**	<10	44 (40.74)	21 (47.73)	22 (50.00)	1 (2.27)
	10–20	23 (21.30)	14 (60.87)	9 (39.13)	0 (0.00)
	>20	41 (37.96)	24 (58.54)	16 (39.02)	1 (2.44)
**Age group**	Children	4 (3.70)	4 (100.00)	0 (0.00)	0 (0.00)
	Children and adults	20 (18.52)	6 (30.00)	13 (65.00)	1 (5.00)
	Adults	84 (77.78)	49 (58.33)	34 (40.48)	1 (1.19)
**Country**	Croatia	3 (2.78)	1 (33.33)	2 (66.67)	0 (0.00)
	Germany	6 (5.56)	4 (66.67)	2 (33.33)	0 (0.00)
	Japan	4 (3.70)	4 (100.00)	0 (0.00)	0 (0.00)
	Poland	81 (75.00)	42 (51.85)	38 (46.92)	1 (1.23)
	United States	8 (7.40)	3 (37.50)	4 (50.00)	1 (12.50)
	Other	6 (5.56)	5 (83.33)	1 (16.67)	0 (0.00)
**Chemical form**	Calcium carbonate	72 (66.67)	41 (56.94)	29 (40.28)	2 (2.78)
	Calcium lactate	9 (8.33)	2 (22.22)	7 (77.78)	0 (0.00)
	Calcium salts of orthophosphoric acid	7 (6.48)	3 (42.86)	4 (57.14)	0 (0.00)
	Tricalcium phosphate	3 (2.78)	1 (33.33)	2 (66.67)	0 (0.00)
	Other forms	6 (5.56)	5 (83.33)	1 (16.67)	0 (0.00)
	Several chemical forms	7 (6.48)	5 (71.43)	2 (28.57)	0 (0.00)
	No declaration	4 (3.70)	2 (50.00)	2 (50.00)	0 (0.00)
**Declaration of the content**	<100	16 (14.81)	11 (68.75)	5 (31.25)	0 (0.00)
	100–200	47 (43.52)	20 (42.55)	27 (57.45)	0 (0.00)
	>200	45 (41.66)	28 (62.22)	15 (33.33)	2 (4.45)
**Type of food supplement**	Single	13 (12.04)	5 (38.46)	8 (61.54)	0 (0.00)
	Complex	95 (87.96)	54 (56.84)	39 (41.05)	2 (2.11)

**Table 9:** Conditions for the mineralization of food supplements.

Steps	Temperature (°C)	Time (min)	Pressure (atm)	Microwave Power (%)
1	170	10	20	80
2	190	10	30	90
3	210	18	40	90
4	50	10	40	0

## Data Availability

Data are available from the authors.

## Sample Availability

Not available.
